# Calcium Transport in Specialized Dental Epithelia and Its Modulation by Fluoride

**DOI:** 10.3389/fendo.2021.730913

**Published:** 2021-08-11

**Authors:** Veronica Costiniti, Guilherme H. Bomfim, Erna Mitaishvili, Ga-Yeon Son, Yi Li, Rodrigo S. Lacruz

**Affiliations:** Department Molecular Pathobiology, College of Dentistry, New York University, New York, NY, United States

**Keywords:** Ca^2+^, fluoride, enamel, ameloblasts, store operated Ca^2+^ entry, amelogenesis imperfecta, fluorosis

## Abstract

Most cells use calcium (Ca^2+^) as a second messenger to convey signals that affect a multitude of biological processes. The ability of Ca^2+^ to bind to proteins to alter their charge and conformation is essential to achieve its signaling role. Cytosolic Ca^2+^ (_c_Ca^2+^) concentration is maintained low at ~100 nM so that the impact of elevations in _c_Ca^2+^ is readily sensed and transduced by cells. However, such elevations in _c_Ca^2+^ must be transient to prevent detrimental effects. Cells have developed a variety of systems to rapidly clear the excess of _c_Ca^2+^ including Ca^2+^ pumps, exchangers and sequestering Ca^2+^ within intracellular organelles. This Ca^2+^ signaling toolkit is evolutionarily adapted so that each cell, tissue, and organ can fulfill its biological function optimally. One of the most specialized cells in mammals are the enamel forming cells, the ameloblasts, which also handle large quantities of Ca^2+^. The end goal of ameloblasts is to synthesize, secrete and mineralize a unique proteinaceous matrix without the benefit of remodeling or repair mechanisms. Ca^2+^ uptake into ameloblasts is mainly regulated by the store operated Ca^2+^ entry (SOCE) before it is transported across the polarized ameloblasts to reach the insulated enamel space. Here we review the ameloblasts Ca^2+^ signaling toolkit and address how the common electronegative non-metal fluoride can alter its function, potentially addressing the biology of dental fluorosis.

## Introduction

Calcium (Ca^2+^) is the third most abundant metal in nature. The chemical properties of Ca^2+^, its radius (0.99 Å), hydration energy and charge, are such that they facilitate its role as a signaling messenger in cells favored over other cations, such as magnesium (Mg^2+^), that have a smaller radius and higher hydration energy (1). Its chemistry also allows Ca^2+^ to optimally accept binding sites of irregular geometry, in contrast with Mg^2+^, which requires octahedral-binding sites that are not commonly found in proteins. Instead, proteins offer a wide range of binding sites for Ca^2+^ (2) and therefore, Ca^2+^ has become a global phenomenon in cell signaling to the point that it is virtually impossible to consider a biological function where Ca^2+^ does not play a role (3). The impact of Ca^2+^ signaling on cell function is closely linked to changes in its concentration within cells. Cytosolic Ca^2+^ (_c_Ca^2+^) concentration is maintained low at ~100 nM compared to ~2 mM outside the cell and therefore, small changes in _c_Ca^2+^ have important effects (3). An excessively high elevation in _c_Ca^2+^ could result in mitochondrial Ca^2+^ overloading, activation of proteases, activation of DNA-fragmenting enzymes and cell death, consequently, elevations in _c_Ca^2+^ must be transient (4). Cells have adopted complex systems to clear excess of Ca^2+^ including pumps, exchangers, Ca^2+^-binding proteins as well as using intracellular organelles such as the endoplasmic reticulum (ER) and mitochondria as Ca^2+^ sinks (5).

Across cells and organs, the involvement of Ca^2+^ in signaling can be limited in scope, such as the elementary events involving Ca^2+^ exchange between the ER and mitochondria or can be more global such as the Ca^2+^ waves associated with muscle contraction (6). Although the signaling toolkit in mammalian cells is highly conserved, the physiological output of these signaling events is logically distinct across cells and organs. In some ways, it would be reasonable to consider that the role of Ca^2+^ in mineralizing cells, i.e. dental enamel and bone cells, would be well understood given their reliance on Ca^2+^ to form these tissues, however, this isn’t the case. The most highly calcified tissue of vertebrates, dental enamel, offers an attractive model for potentially decoding relevant Ca^2+^ signatures in mineralizing systems, yet knowledge of the identity and function of the Ca^2+^ toolkit of the enamel forming cells remains in its infancy. In this review, we will explore the most relevant aspects of what is known about Ca^2+^ transport in enamel cells, the ameloblasts, and will focus on the effects of a worldwide phenomenon caused by excessive amounts of another metal, fluoride, and how these two metals converge in a disease known as dental fluorosis.

## Ca^2+^ in Enamel

Ca^2+^ is a key element in the composition of dental enamel (7), being required during the two stages of enamel development, namely the secretory and maturation stages. In the secretory stage, thin enamel crystals elongate within an organic matrix template formed by key enamel structural proteins (e.g. amelogenin, ameloblastin and enamelin) in a highly organized fashion (7). During the maturation or mineralization stage, crystals expand in width and thickness as the organic matrix is removed. Ca^2+^ requirements increase at this stage (7). Ameloblasts are epithelial cells of ectodermal origin that form and mineralize enamel. A mention of their morphological characteristics is relevant because secretory ameloblasts are ~65 µm in height and maturation are ~35 µm and both maintain a narrow diameter of ~5 µm. Therefore, ameloblasts are highly polarized cells and form a tight cell barrier, limiting intercellular passage of ions and minerals (7). The possibility of a passive Ca^2+^ transport system across the interstitial space is an unlikely scenario given the presence of tight junctions adjoining the distal pole of maturation stage ameloblasts (8). Passive transport also appears counterintuitive because the highly organized nature of the mineralized enamel crystals suggests a non-haphazard stoichiometric accumulation of ions in the enamel fluid (9).

Ameloblasts not only use Ca^2+^ as a signaling messenger, of which we have limited understanding, they must also transport Ca^2+^ safely across the cell. Hubbard indicated that the transcellular Ca^2+^ transport in ameloblasts is complex and requires an understanding of several steps (10): ***a)*** entry, ***b)*** transit and ***c)*** extrusion. For a very long time, the molecular pathways regulating these steps in ameloblasts had remained undefined. Recently, several studies of human mutations and rodent models indicate that the store operated Ca^2+^ entry (SOCE) pathway is essential for enamel mineralization, being the primary Ca^2+^ uptake systems in ameloblasts (11–13).

## Store Operated Ca^2+^ Entry in Ameloblasts

SOCE, also known as Ca^2+^ release activated Ca^2+^ (CRAC) channels, is an essential and widely expressed Ca^2+^ influx channel to the extent that SOCE dominates the ability of non-excitable cells to uptake Ca^2+^ in physiological and pathological conditions (14). CRAC channels are formed by the Ca^2+^ sensors STIM1 and 2 (stromal interaction molecules) found in the membrane of the ER, and the channel pore formed by the ORAI proteins in the plasma membrane (ORAI1-3) (14–16). CRAC channels are activated following the stimulation of a cell surface receptor resulting in the production of PLC (phospholipase-C) and InsP3 (inositol 1,4,5 triphosphate), which in turns binds to its ER membrane receptors, the IP_3_R (17, 18). InsP_3_-IP_3_R interactions elicit the release of ER Ca^2+^ pools *via* the receptor channel (18, 19). A decline in luminal Ca^2+^ concentration in the ER triggers substantial conformational changes in STIM proteins leading to the binding and activation of the ORAI channel to allow a sustained Ca^2+^ influx (14, 20). Mutations in *STIM1* or *ORAI1* genes cause channelopathy including immune dysfunction and ectodermal dysplasia, amelogenesis imperfecta (AI), muscle weakness and anhidrosis (21, 22).

The links between SOCE and AI became evident in a series of papers published by the Feske laboratory reporting that patients with mutations in *ORAI1* or *STIM1* showed clear enamel defects described as type 2 hypomineralized AI (23, 24). Similar findings have been reported by other groups (25). Our studies using CRAC channels inhibitors such as synta-66, BTP-2 and GSK7975A, in rat ameloblasts, showed that these blockers markedly reduced or nearly abolished Ca^2+^ influx *via* SOCE (13, 26). We also showed that mice lacking STIM1/2 or ORAI1, which exhibited deficient SOCE in ameloblasts, had enamel defects raging from severe hypomineralization to disruptions in enamel crystal formation (11, 12). Combined, the human and mouse data highlight the critical role of SOCE as a key Ca^2+^ influx channel in enamel cells.

## Potential Soce Modulators in Ameloblasts

Although STIM1/2 and ORAI1-3 proteins are the core components of SOCE, a number of molecular modulators have been described having various effects on SOCE, reviewed in (27). These include proteins that stabilize STIM–ORAI interactions, stimulate STIM1 conformational changes or induce slow Ca^2+^-dependent inactivation (27). A recent study reported that mutations in the solute carrier *SLC10A7* gene results in hypomineralized enamel and AI (28). A more recent study suggested that SLC10A7 is a negative modulator of SOCE, because the knockdown of *SLC10A7* resulted in increased SOCE (29). The mechanism of the interaction between SLC10A7 and SOCE is unknown, although some possibilities have been proposed: 1) disrupts sarcoendoplasmic reticulum calcium transport ATPase (SERCA) function, 2) destabilizes STIM1 oligomers or 3) interferes with ORAI. Based on human and mouse studies, a possible cause of the enamel defects caused by *SLC10A7* mutations was associated with a deficiency in glycosaminoglycan (GAG) synthesis (28). GAGs are important components of extracellular matrix and when GAG degradation pathways are disrupted due to enzyme deficiency, GAGs accumulate causing skeletal dysplasia, and could also be the cause of enamel defects (30). However, this novel connection between SOCE and SLC10A7 suggests that Ca^2+^ could also play a role.

Another negative modulator of SOCE, known as SARAF (SOCE-associated regulatory factor), may be an important factor in SOCE modulation in ameloblasts. SARAF (also known as TMEM66) is an ER membrane protein that associates with STIM to promote Ca^2+^ dependent inactivation of SOCE (31). We found that *Saraf* was significantly upregulated in maturation stage ameloblasts relative to secretory ameloblasts (32), suggesting a possible function in enamel but no data is currently available.

Of significance are recent reports on *Trpm7*-inactive knock-in mutant mice which showed hypomineralized enamel in the heterozygous mice (33). The dental phenotype, as well as, other skeletal anomalies were ascribed to Mg^2+^ deficiency, which was required for alkaline phosphatase activity and mineralization (33). The transient receptor potential melastatin 7 (TRPM7) had been characterized as an ion channel permeable to divalent cations (Mg^2+^, Ca^2+^) linked to an intrinsic kinase domain, enabling it to modulate cellular functions (34). Faouzi et al. reported that although they did not consider TRPM7 a SOCE component, its kinase domain had a modulatory effect on SOCE (35). Our investigations into the role of TRPM7 in primary ameloblasts showed that TRPM7 potentiates Ca^2+^ influx *via* SOCE, and reported that its function is fully dependent on the prior activation of the ORAI1 channels (36).

## Ca^2+^ Transit and Removal in Ameloblasts

Hubbard’s original groundbreaking studies on the identification of several Ca^2+^ proteins in ameloblasts and their upregulation in maturation (37–40), led him to suggest that Ca^2+^ may be transiting the ameloblasts *via* a type of safe tunneling mechanism which might implicate the ER tubules, a model he termed the “transcytosis” model (10, 41). The lack of enamel phenotype in mice with disrupted function of the Ca^2+^ binding proteins known as calbindins, reinforced this model and appeared to rule out a possible scenario where calbindins could ferry Ca^2+^ across the ameloblasts (42). Besides calbindins, ameloblasts express several other Ca^2+^ buffering proteins (8). However, how Ca^2+^ may be reaching the distal pole before it is extruded out of the cell, remains a significant gap in knowledge in enamel biology.

As highlighted above, it is important that elevations in _c_Ca^2+^ concentrations are transient. To control this, cells employ two efficient systems to remove the Ca^2+^ from the cytosol: Ca^2+^ pumps and Ca^2+^ exchangers (3, 4, 43, 44).

Plasma membrane Ca2+-ATPases (PMCAs), or Ca^2+^ pumps, found in the cell membranes, that translocate Ca^2+^ from the inside to the outside of the cell, consuming ATP in the process (2, 45). PMCAs have high affinity for Ca^2+^ (K_d_ ~0.1 mM) but have low transport capacity, pumping 1 Ca^2+^ per ATP consumed (2, 45, 46). PMCAs are coded by the *ATP2B* genes and three of these genes (*ATP2B*1,3 and 4) are expressed in enamel cells, appearing to be upregulated during the secretory stage (47). However, whether PMCAs are functional in ameloblasts or whether there are differences across stages, has not been reported to date.

Ca^2+^ exchangers are also important in removing _c_Ca^2+^ out of the cell and unlike PMCA, the exchangers do not require ATP hydrolysis. Instead, the Na^+^/Ca^2+^ exchangers use the chemical energy of the Na^+^ gradient (the Na^+^ concentration is much higher outside of the cell than inside the cell) to remove Ca^2+^ from the cytosol (3, 4). The exchangers have low Ca^2+^ affinity but high transport capacity with estimated values of ~5000 Hz for NCX (48). Because their function depends on electrochemical driving forces across the plasma membrane, the exchangers can operate in a forward mode (Ca^2+^ extrusion mode), which is their physiological function, as well as, in a reverse mode (Ca^2+^ uptake) (49).

There are two main families of Ca^2+^ exchangers, the NCX and NCKX. The NCX are Na^+^/Ca^2+^ exchangers in the plasma membrane, coded by the *SLC8A* genes, that remove 1 Ca^2+^ in exchange for 3 Na^+^ (49). By contrast, the NC**K**X family of proteins, coded by *SLC24A* genes, remove 1 Ca^2+^and 1 K^+^ in exchange for 4 Na^+^ (50). Unlike the NCX family, NCKXs require K^+^ to accomplish the Ca^2+^ exchange (50, 51). NCX were reported originally in ameloblasts in an outstanding paper by Okumura and colleagues (52). However, to date, there are no known mutations in the *SLC8A* genes causing AI. It was probably our identification of NCKX4 in ameloblasts (32, 53, 54) that improved our knowledge on the proteins having an essential role in Ca^2+^ extrusion in ameloblasts. We and others have suggested that NCKX4 is important in enamel mineralization (25, 54) because the expression of NCKX4 in maturation is high, the highest of the six NCKX family members in ameloblasts (54), and because mutations in the coding gene (*SLC24A4*) in humans and mouse models results in enamel defects (25, 55). Its localization in the distal pole of maturation ameloblasts would be consistent with a role in Ca^2+^ extrusion likely being more prominent in maturation (54). However, there are no currently published data on the functionality of NCKX4, and no data in maturation stage ameloblasts for NCX.

## Disruptions on Ca^2+^ Homeostasis: The Case of Fluorosis

Having briefly described the broader picture of the Ca^2+^ handling system in ameloblasts, an interesting case study linking an enamel disease and disruptions in Ca^2+^ homeostasis is represented by dental fluorosis, a disease that arises when excessive amounts of fluoride are ingested during childhood, the time during which enamel development takes place (56, 57). The effects of fluoride on enamel are dose-dependent (58, 59) and result in pitted or discolored enamel prone to fracture, and increased wear and caries (56, 58). In chronic situations, fluorosis can lead to skeletal dysfunction causing bone breakage (58). The prevalence of dental fluorosis varies across countries. In the US, ~41% of adolescent population showed varying degrees of fluorosis (60). In India, dental fluorosis has a major impact with estimates of ~62 million people being affected (61). Clearly, dental fluorosis remains a world health issue, and despite decades of research, the proximate causes remain unclear.

## Effects of Fluoride on Enamel

The benefits of fluoride in caries prevention have been known for decades. Therefore, the controlled supplementation of fluoride in drinking water or table salts has become a common practice in many countries. In the US, the most recent recommended concentration of fluoride in drinking water is ~0.7 parts per million (ppm), providing the best balance of protection from dental caries while limiting the risk of dental and/or skeletal fluorosis. At this concentration, fluoride has a positive effect on enamel strengthening chemical bonds in the formed enamel crystals, decreasing the risk of caries once the enamel crown has erupted (62–64). Excessive consumption of fluoride however leads to retention of enamel matrix proteins, e.g. amelogenin, and hypomineralization (58, 64). The effects on ameloblasts are stage-dependent with differences observed in the secretory and maturation stages (62–64). In the secretory stage, excess of fluoride inhibits protein secretion whereas in maturation it disrupts the cyclic modulation of ruffled-to-smooth ended ameloblasts which is important for ion transport (58, 59, 65). It has been reported that the ameloblast cell line LS8 treated with ≥1 mM concentrations of fluoride (NaF), results in ER stress and **u**nfolded **p**rotein **r**esponse (UPR) (66–69). This evidence suggests that excess of fluoride affects ameloblast biology, which could result in disruptions in crystal formation. Bronckers and colleagues suggested that fluoride causes hypermineralization of the enamel resulting in an increase in the proton load, a byproduct of crystal formation (65). Interestingly, they propose that this hypermineralization effect results in a barrier that prevents proper ion transport and protein recycling causing the hypomineralization effect associated with dental fluorosis (65).

One consideration that remains unclear is how fluoride access the ameloblasts. Fluoride could plausibly cross the ameloblast membranes *via* diffusion as hydrogen fluoride (HF), as reported in other cells (70). However, we have previously raised the possibility that it crosses the ameloblasts’ membrane *via* chloride channels, given their expression in ameloblasts (53, 71). Currently, the cellular uptake pathway of fluoride in enamel cells is not clear.

## Interactions of Fluoride and Ca^2+^ Homeostasis

**Figure 1:**
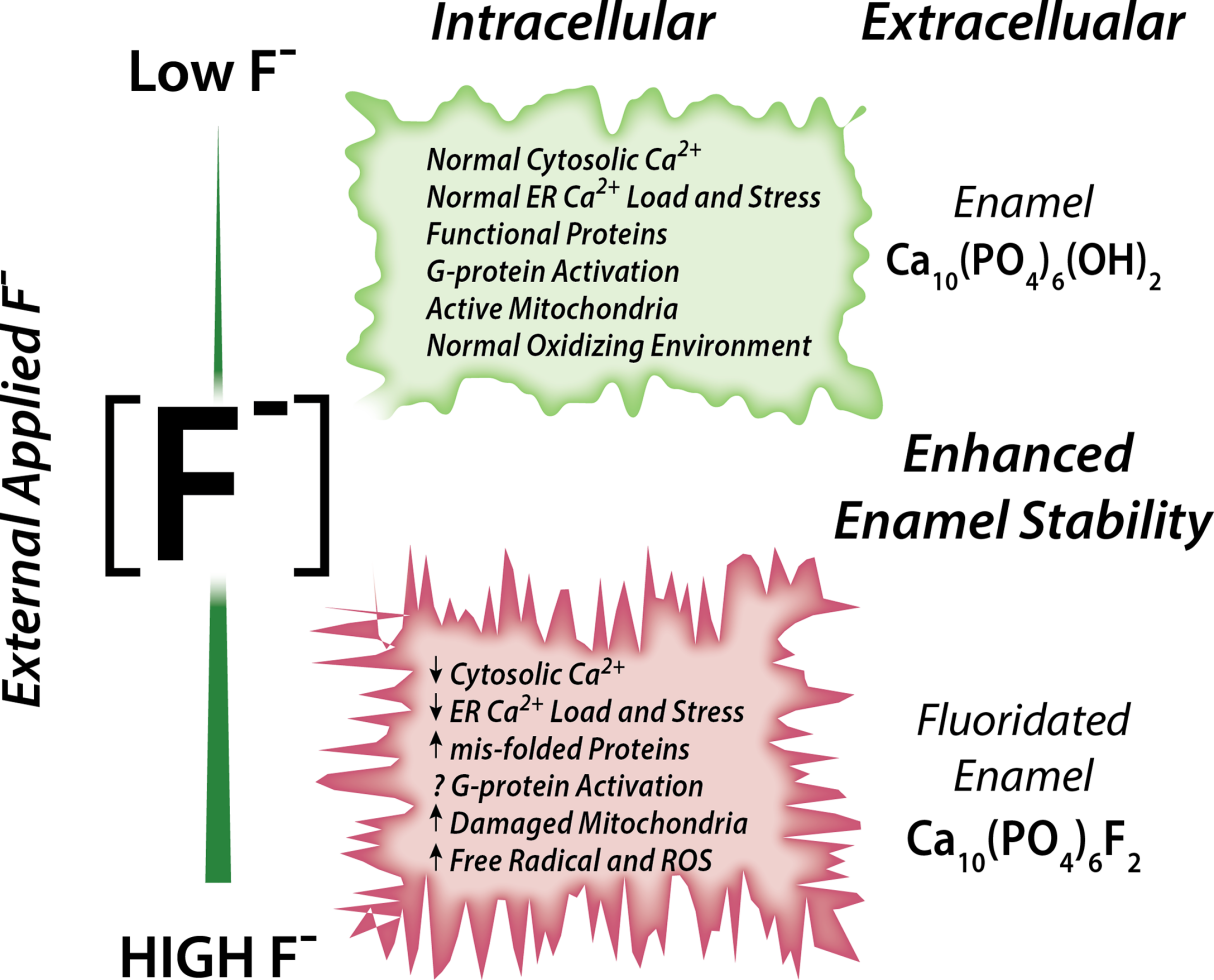
Schematic of the effects of fluoride in enamel cells. Fluoride modulates Ca^2+^ homeostasis with a dual effect. Low concentration leads to normal cytosolic and ER Ca^2+^ load, lack of ER stress leading to normal ER and mitochondrial function. High fluoride induces abnormal cytosolic and ER Ca^2+^, possibly *via* impairment of G-protein activation, and dysregulates cell metabolism.

Associations between fluoride and Ca^2+^ have been reported (70). Rats drinking fluoridated water also receiving a dietary Ca^2+^ supplement showed ameliorated negative effects in their bones and kidney (analyzed histologically) than rats drinking the same fluoridated water but *without* receiving a Ca^2+^ supplement, strongly suggesting that Ca^2+^ has a protective role in fluorosis toxicity (72). It is also noteworthy that acute NaF exposure in rats resulted in hypocalcemia in plasma (73). Experiments conducted in osteoblasts and in rat proximal tubule showed that, in the presence of external Ca^2+^, fluoride stimulation at 10 µM concentration results in a rapid elevation of _c_Ca^2+^ (74, 75). These elevations in _c_Ca^2+^ were likely mediated by activation of G-protein-coupled receptors (GPCRs) (74, 75). These reports support the notion that fluoride modulates Ca^2+^ homeostasis in cells, as summarized in **Figure 1**.

## The Disruptive Effects of Fluoride on Ameloblast Ca^2+^ Signaling

**Figure 2:**
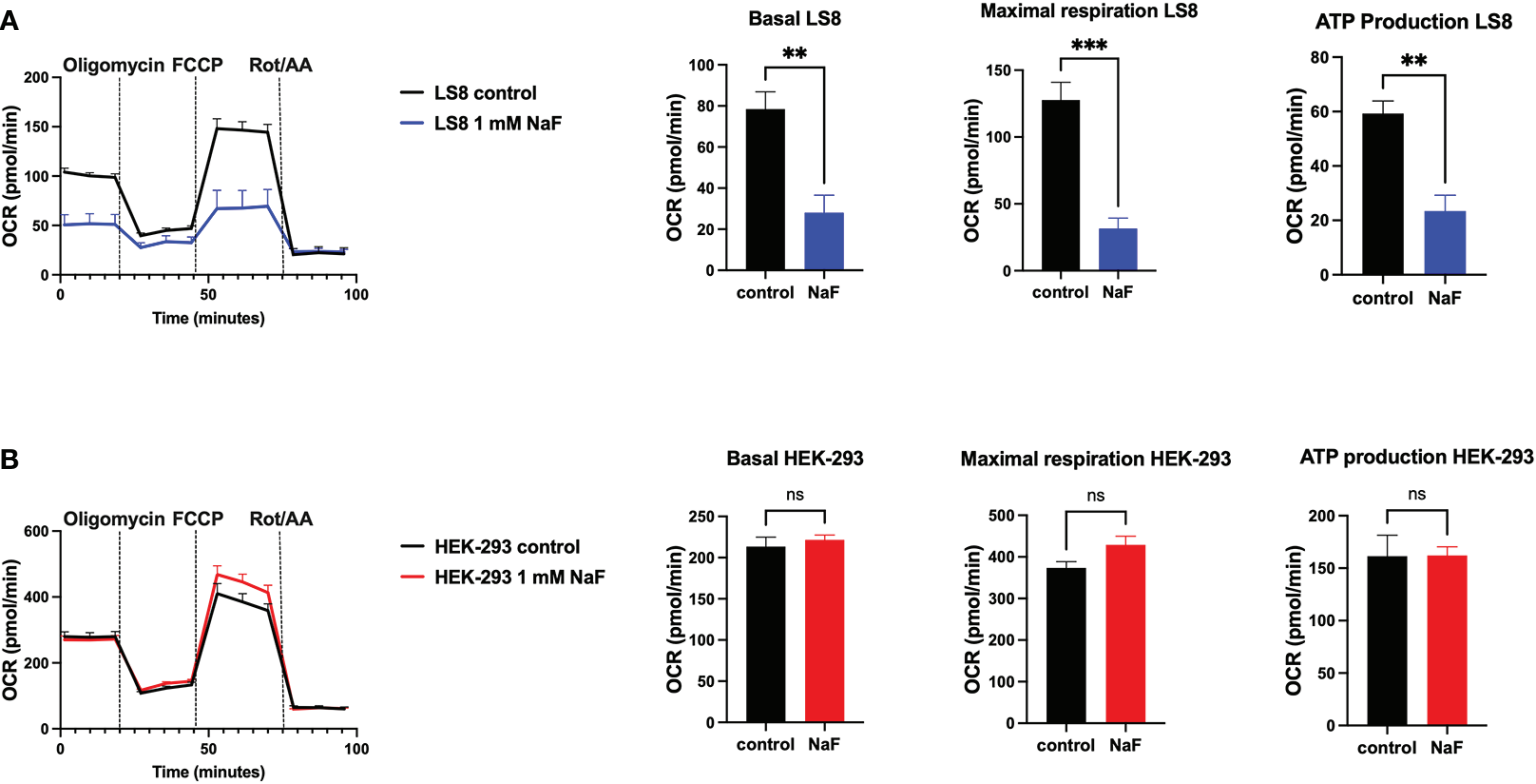
Mitochondrial OCR in fluoride treated cells. Oxygen consumption rate (OCR), basal respiration, ATP production and maximal respiration in LS8 cells **(A)** and in HEK-293 cells **(B)** after 4 hours of NaF (1 mM) pre-treatment. Oligomycin (1 µM), FCCP (1.5 µM) and rotenone/antimycin A (Rot/AA - 0.5 µM) were serially added in a Seahorse XFe24 Analyzer to assess differences in oxidative phosphorylation. Fluoride treatment affects OCR in the enamel LS8 cells but not HEK-293 cells (ns, not significant). Data represent the mean ± SEM of 3 independent experiments using unpaired Student’s t test. (**p < 0.01, ***p < 0.001).

A recent study using rat primary ameloblasts and LS8 cells exposed to various concentrations of fluoride *in vitro* showed decreased internal Ca^2+^ in the ER and SOCE (76). These effects were observed when using high NaF concentrations (0.5 mM, 1 mM), equivalent to ~9 ppm and 18 ppm, respectively. In primary ameloblasts, these fluoride treatments decreased the ER Ca^2+^ within 30 minutes of incubation, and resulted in ER stress (76). LS8 cells exposed to bromide (NaBr) did not change these functions. An unexpected finding of the study was that treating LS8 and HEK-293 cells with the same concentration (1 mM) of NaF in similar conditions did not affect ER Ca^2+^ or SOCE in the latter. Moreover, NaF affected mitochondrial function in LS8 cells. Because this was not investigated in HEK-293 cells, we performed the seahorse mitochondrial assay like that was reported by Aulestia et al. in LS8 cells. Results show that while mitochondrial respiration in LS8 cells was negatively affected by NaF (1 mM) (**Figure 2A**), HEK-293 cells were not (**Figure 2B**) supporting the notion that mineralizing cells might be more sensitive to fluoride than other cells.

The fluoride concentrations used above are relatively high and mimic the effects of fluorosis. However, we found that fluoride, at lower dosage, also had a negative effect on Ca^2+^ signaling in LS8 cells. When LS8 cells were treated for 24 hours with 10 µM of NaF, equivalent to ~0.2 ppm, it disrupted the function of the ER-localized Ca^2+^ channel IP_3_R and the activity of the SERCA pump during Ca^2+^ refilling of the ER (76). These data, we believe, provide a mechanism that can potentially address the biology of dental fluorosis or, at the very least, provide important information on the effects of fluoride in ameloblast Ca^2+^ physiology.

## Summary

Enamel is a prototypical example of biologically controlled mineralization. Ameloblasts form a boundary that encloses the space of mineral formation and have the ability to control the introduction of ions into that space. The introduction of Ca^2+^ is essential for the mineralization of the long and thin hydroxyapatite enamel crystals. SOCE is the dominant system controlling the uptake of Ca^2+^ in ameloblasts. As Ca^2+^ crosses the ameloblast’s membrane, SERCA helps weather the effects of the Ca^2+^ storm but surely other mechanisms must be in place to ensure that ameloblasts protect cell function. However, little is known about these other processes including the capacity of mitochondria to uptake Ca^2+^ or the practicability of the clearance mechanisms by pumps and exchangers. Such information would be important to address the ins and outs of how enamel is mineralized by the ameloblasts. At any rate, it would appear that several components of the Ca^2+^ signaling toolkit of the ameloblasts are hindered by fluoride altering their physiology and function, likely affecting enamel mineralization.

## Author Contributions

All authors contributed to the article and approved the submitted version.

## Funding

This work was funded by NIH/National Institute of Dental and Craniofacial Research (NIDCR) awards (DE025639 and DE027679) to RL.

## Conflict of Interest

The authors declare that the research was conducted in the absence of any commercial or financial relationships that could be construed as a potential conflict of interest.

## Publisher’s Note

All claims expressed in this article are solely those of the authors and do not necessarily represent those of their affiliated organizations, or those of the publisher, the editors and the reviewers. Any product that may be evaluated in this article, or claim that may be made by its manufacturer, is not guaranteed or endorsed by the publisher.
